# Emerging roles of lipid transfer protein dimerization

**DOI:** 10.1242/jcs.263971

**Published:** 2025-08-08

**Authors:** Shalom Borst Pauwels, Jacques Neefjes, Birol Cabukusta

**Affiliations:** Cell and Chemical Biology, ONCODE Institute, Leiden University Medical Center, 2333ZA Leiden, Netherlands

**Keywords:** Lipid transfer proteins, Non-vesicular trafficking, Membrane contact sites, Dimerization

## Abstract

The unique lipid composition of organelles defines their identity and is fundamental to their function. Lipid transfer proteins perform non-vesicular trafficking of lipids among cellular membranes to maintain their lipid compositions. Lipid transfer protein-mediated lipid trafficking is also essential for creating sub-organellar nano-domains that can recruit functional proteins or change the biophysical properties of membranes. The latest research focusing on the homo- and hetero-dimerization of lipid transfer proteins highlights the functional implications and the clinical significance of these events. Dimerization promotes lipid transfer protein localization at membrane contact sites and mediates the assembly of lipid transfer protein super-complexes to synchronize the transfer of different lipid types between organelles. Meanwhile, abnormal lipid flows caused by disarrangements in lipid transfer protein dimerization disturb organelle lipid landscapes, which has clinical consequences. This Review discusses the latest developments regarding the dimerization of lipid transfer proteins and their adaptor proteins that are critical for lipid trafficking between the organelles of the cell.

## Teaser

**Summary:** Dimerization of lipid transfer proteins is crucial for the localization, regulation and synchronization of lipid trafficking that defines organelle lipid landscapes and cellular fate.

## Introduction

Many types of lipids are heterogeneously distributed among the membrane-bound organelles of eukaryotic cells, and the unique lipid composition of each organelle is essential for its function and identity (Holthuis and Menon, 2014; van Meer and de Kroon, 2011). Even at the sub-organellar level, lateral segregation facilitated by lipids is responsible for creating nanodomains and hotspots that support protein functions (Harayama and Riezman, 2018; Levental and Lyman, 2023). Creating, maintaining and adapting cellular membranes composed of diverse lipid types is an immense task that cells perform continuously.

The hydrophobic nature of lipids requires facilitated trafficking between subcellular compartments. Vesicular transport, which carries lipids in bulk across the secretory and endocytic pathways, cannot account for the vast variety of lipid trafficking routes cells demand. Organelles outside of these vesicular routes, such as mitochondria, lipid droplets and peroxisomes, do not receive vesicular cargo and therefore require alternative mechanisms for lipid exchange. Lipid transfer proteins (LTPs) use their hydrophobic cavities to transport lipids independently of vesicular trafficking, a mechanism known as non-vesicular transport (Wong et al., 2019). For this purpose, LTPs often localize to membrane contact sites, regions where two organelles are in close apposition. The evolutionary emergence of LTPs can be dated as early as gram-negative prokaryotes, which require lipid transport between their inner and outer cell membranes (Levine, 2022). In eukaryotes, numerous LTPs are classified based on the homology of their lipid transfer domains.

LTPs can transport lipids at rates unmatched by vesicular trafficking. This capability is especially advantageous when organelles need to swiftly alter their lipid composition in response to stimulation. For example, LTPs situated between the endoplasmic reticulum (ER) and the plasma membrane supply lipids to the plasma membrane within minutes, bypassing the secretory pathway, which can take up to an hour (Boncompain et al., 2012; Lees et al., 2017). Furthermore, LTPs can deliver lipids to organelle subdomains, increasing the local concentration of bioactive lipids, which can change the biophysical properties of membranes or recruit proteins to these subdomains (Guyard et al., 2022; Kawasaki et al., 2022; Mesmin et al., 2017; Tan and Finkel, 2022). The ability of LTPs to rapidly spike local lipid concentrations has made them an attractive focus of research over the past few decades (Johnson et al., 2024; Kim et al., 2024).

**Fig. 1.**
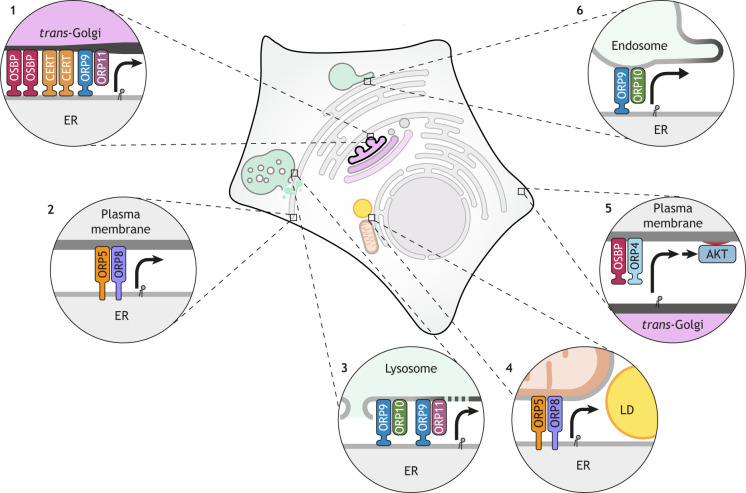
**Overview of LTP dimers shaping organelle lipid landscapes and creating membrane nano-domains enriched in bioactive lipids.** (1) The OSBP and CERT homodimers, along with the ORP9–ORP11 heterodimer, deliver lipids from the ER to the *trans*-Golgi, establishing plasma membrane identity and transforming membrane thickness. (2) The ORP5–ORP8 heterodimer localizes at ER–plasma membrane contact sites to exchange lipids. (3) The ORP9–ORP10 and ORP9–ORP11 heterodimers provide lipids to damaged lysosomes for membrane repair. (4) The ORP5–ORP8 heterodimer localizes to ER–mitochondria–lipid droplet (LD) tripartite contact sites to coordinate lipid droplet biogenesis. (5) Ectopic expression of the OSBP–ORP4 heterodimer initiates plasma membrane lipid signaling, leading to tumorigenesis. (6) The ORP9–ORP10 heterodimer provides lipids for endosomal protrusion and fission.

One of the latest research highlights has been the recognition of the functional implications and clinical significance of LTP homo- and hetero-dimerization. Although some LTP dimers had been identified previously, it is only recently that the relevance of these events become clear. Although the current atomic models of these interactions are mostly hypothetical, this emerging understanding transforms our perspective on how LTPs regulate lipid flows between organelles. In this Review, we focus on LTPs and their adaptor proteins that require homo- and hetero-dimerization for their function in regulating and maintaining lipid trafficking across the organelles of the cell. While the lipid transfer functions of these proteins, particularly oxysterol-binding protein (OSBP) and ceramide transfer protein (CERT; also known as CERT1), have been reviewed elsewhere (Hamai and Drin, 2024; Mizuike and Hanada, 2024; Subra et al., 2023a; Wong et al., 2019), this Review focuses on the fundamental mechanisms, and the cell biological and current clinical implications of dimerization events (Fig. 1).

## Homotypic LTP dimers at ER–*trans*-Golgi contact sites

The ER executes the majority of lipid synthesis and provides lipids to other organelles and the plasma membrane (Balla et al., 2020; Holthuis and Menon, 2014; Wenzel et al., 2022). Phosphatidylserine (PS), cholesterol and sphingolipids produced in the ER are enriched on the plasma membrane, whereas their levels in the ER are kept low. The ER forms contact sites with the *trans* cisternae of the Golgi, bypassing the *cis*-Golgi despite its physical proximity. This ensures that vesicles budding from the *trans-*Golgi towards the plasma membrane maintain their proper lipid composition (David et al., 2021; Duran et al., 2012; Masone et al., 2019). As secretory proteins undergo essential modifications, such as glycosylation, as they travel through the Golgi stacks, they are sorted to vesicles with a lipid composition best suited for the plasma membrane. The lipid composition of these vesicles is coordinated by LTP dimers at the ER–*trans*-Golgi interface.

### OSBP

**Fig. 2.**
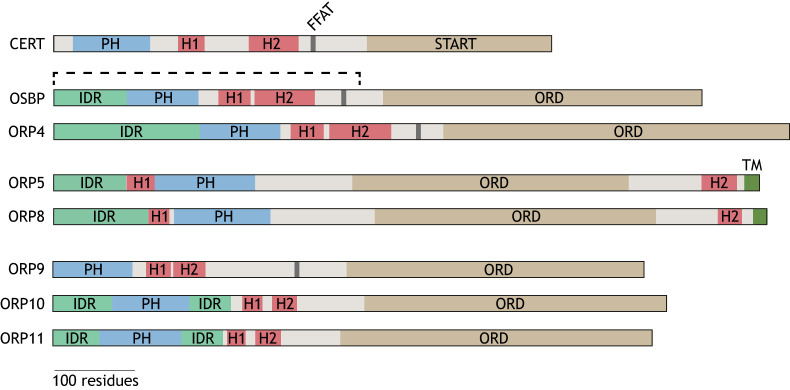
**Domain map of LTPs containing coiled coils for dimerization.** CERT contains a short (H1) and a long (H2) α-helix between its PH and START domains for homodimerization. OSBP and ORP4 both have an N-terminal IDR that limits protein density on the membrane, and a short (H1) and a long (H2) α-helix for dimerization. ORP5 and ORP8 contain an N-terminal IDR, two short α-helices (H1 and H2) and a C-terminal transmembrane helix (TM) for ER anchoring. ORP9, ORP10 and ORP11 have two short α-helices (H1 and H2) that mediate heterodimerization. ORP10 and ORP11 also have IDRs flanking their PH domain. FFAT motifs, as found in CERT, OSBP, ORP4 and ORP9, mediate interaction with ER-resident VAP proteins. The structurally resolved fragment of OSBP is indicated with a dashed bracket.

Cholesterol in the ER, whether newly synthesized or internalized from the extracellular environment, is transported to the *trans*-Golgi by OSBP. The multimeric state of OSBP was established when it was first identified as a cytosolic 25-hydroxycholesterol-binding protein (Dawson et al., 1989b; Taylor and Kandutsch, 1985). Cloning of the OSBP cDNA by the group of Brown and Goldstein revealed two features in the amino acid sequence: an N-terminal glycine- and alanine-rich region preceding its pleckstrin homology (PH) domain, and a leucine zipper, initially thought to be responsible for dimerization (Fig. 2) (Dawson et al., 1989a). OSBP can still dimerize without the leucine zipper, although removing a larger region containing two α-helices diminishes its dimerization (Ridgway et al., 1992).

**Fig. 3.**
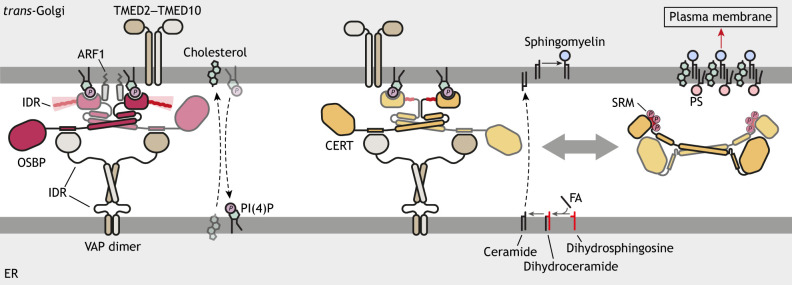
**LTP homodimers at ER–*trans*-Golgi membrane contact sites facilitate the transition from the lightly packed ER membrane to the thicker and denser plasma membrane.** Left, the OSBP dimer interacts with PI(4)P, ARF1 and TMED2–TMED10 for Golgi localization, and with VAP proteins for ER localization. The N-terminal IDR of OSBP limits the protein density on the membrane to maintain dynamic localization of the protein for cholesterol and PI(4)P exchange. Middle and right, the CERT dimer localizes to the contact site in a similar fashion. CERT contains an SRM, phosphorylation of which inhibits CERT by inducing a conformational change. CERT transfers ceramide, which is used for sphingomyelin synthesis in the Golgi. As cholesterol and sphingomyelin form a complex, lipids trafficked at the ER–*trans*-Golgi contact site define the transition from the lightly packed, thin ER membrane to the thicker, denser plasma membrane. In CerTra syndrome, hyperactivated CERT delivers the ceramide precursor dihydroceramide to the Golgi for complex sphingomyelin production.

The architecture of the OSBP homodimer was only recently mapped (de la Mora et al., 2021; Mesmin et al., 2013). In this structure, based on a truncated version of the protein lacking the lipid transfer domain (Fig. 2), the short (H1) and long (H2) α-helices from both OSBP subunits assemble in an anti-parallel fashion to create a three-stranded coiled coil (Fig. 3). Together with the preceding PH domains attached to the small GTPase ARF1 and phosphatidylinositol 4-phosphate [PI(4)P] on the membranes, the dimer adopts a ‘rigid’ T-shaped structure. This rigidity provides structural stability, while the arms of the T remain sufficiently flexible to interact with vesicle-associated membrane protein (VAMP)-associated proteins (VAPA and VAPB) on the ER and efficiently transfer lipids between membranes.

Further studies have revealed the function of the N-terminal glycine- and alanine-rich region (Jamecna et al., 2019). This intrinsically disordered region (IDR) increases the hydrodynamic radius, i.e. the effective size of OSBP, which in turn limits its density on the Golgi surface (Fig. 3). The sparse distribution of the protein on the membrane surface allows for mobility within the crowded and narrow membrane contact site, maintaining the dynamic composition of these regions. Conversely, without the IDR, OSBP density on the Golgi surface increases, and the protein is unable to dissociate, preventing its recycling. In addition to facilitating protein mobility, the IDR promotes efficient protein orientation at membrane contact sites, favoring heterotypic tethering between the ER and Golgi over homotypic tethering between two PI(4)P-containing Golgi membranes.

Similar N-terminal IDRs are found in OSBP-related proteins (ORPs). The N-terminal IDR of ORP4 (also known as OSBP2), also rich in alanine, glycine and proline residues, increases its hydrodynamic radius (Fig. 2) (Jamecna et al., 2019). The long variant of ORP8 (also known as OSBPL8), called ORP8L, has an IDR enriched with negatively charged amino acids, rendering the protein less efficient in localizing to the plasma membrane compared to the short ORP8 variant, ORP8S, and its closest homolog ORP5 (also known as OSBPL5) (further discussed below) (Chung et al., 2015).

### CERT

CERT traffics newly synthesized ceramide from the ER to the *trans-*Golgi, where it is converted into sphingomyelin. Structural modeling of CERT has revealed that it has two α-helices between its PH domain and the lipid-trafficking steroidogenic acute regulatory protein-related lipid transfer (START) domain (Fig. 2) (Gehin et al., 2023). Similar to OSBP, CERT dimerizes via an interface formed by a short and a long α-helix that is predicted to come together in an anti-parallel orientation to adopt a T-shaped structure (Fig. 3).

Interestingly, CERT is also reported to form homotrimers, and its extracellular form has been observed as a tetramer and higher oligomer as well (Charruyer et al., 2008; Goto et al., 2022; Raya et al., 2000; Revert et al., 2018). The question of why CERT forms multiple types of self-assemblies is yet to be addressed. This discrepancy in observed self-assemblies might be explained by the different methods used to investigate CERT assembly, i.e. bacterial versus eukaryotic expression systems that reveal dimeric and trimeric forms, respectively (Gehin et al., 2023; Revert et al., 2018). It remains to be elucidated under which conditions CERT forms dimers or trimers and whether the same interface participates in forming CERT trimers.

CERT activity is regulated by phosphorylation, which is thought to induce a conformational change in the homodimer and homotrimer (Fig. 3) (Gehin et al., 2023; Goto et al., 2022). The serine-repeat motif (SRM), located downstream of the PH domain, contains multiple phosphorylation sites. Hyperphosphorylation of this region causes an autoinhibitory conformational change, downregulating lipid transfer activity (Kumagai et al., 2007; Prashek et al., 2017; Sugiki et al., 2018). The phosphorylated SRM and START domain occupy the surface of the PH domain, which is reserved for PI(4)P binding, rendering both domains inactive and unable to link the ER to the Golgi for ceramide transfer.

Disrupted autoregulation of CERT activity causes intellectual disability, known as CerTra syndrome (Gehin et al., 2023). In CerTra syndrome, overactive CERT overfeeds sphingomyelin synthesis. As a result, glycosphingolipid production in the Golgi, which relies on ceramide delivered by vesicular trafficking, is reduced. Furthermore, overactive CERT competes with ceramide desaturase activity in the ER by transferring dihydroceramide to the *trans-*Golgi, leading to excessive production of dihydrosphingomyelin. Both impaired glycosphingolipid synthesis and elevated dihydrosphingolipid levels have been associated with various neurological disorders, possibly explaining the symptoms observed in CerTra syndrome (Bhuiyan et al., 2019; Boccuto et al., 2014; Tzou et al., 2023).

Several CerTra-associated variants are found in the SRM, reflecting the importance of this region in CERT regulation. The S135P mutation within the SRM results in overactive protein by disrupting CERT phosphorylation (Murakami et al., 2020). Although many CerTra variants are prevalent in the SRM, others are found at the dimerization interface, suggesting that dimerization is also involved in CERT activity (Gehin et al., 2023; Tamura et al., 2021). These mutations impair the inactivating conformational change upon SRM phosphorylation (Gehin et al., 2023). Moreover, a cluster of basic amino acids located on the surface of the dimerization interface is thought to mediate conformational change by interacting with the negatively charged hyperphosphorylated SRM (Goto et al., 2022).

## LTP heterodimers functionalize organelle membranes

Not all LTPs homodimerize; some form heterodimers with other LTPs. Heterodimerization provides LTPs with localization to organelles they would not engage with intrinsically. As this is fundamental to LTP function in defining organelle lipid compositions, aberrant heterodimerization can disrupt lipid signaling, leading to significant clinical consequences.

### ORP5–ORP8

The identification of a subgroup of ORPs – ORP5, ORP8, ORP9, ORP10 and ORP11 (also known as OSBPL5, OSBPL8, OSBPL9, OSBPL10 and OSBPL11, respectively) – as PS transfer proteins was a significant advance in the study of LTPs (Maeda et al., 2013). ORP5 and ORP8 are present at ER–plasma membrane contact sites where they mediate PI(4)P and PS exchange (Chung et al., 2015). They each contain a single transmembrane helix for ER localization, where PS is synthesized (Fig. 2). ORP5 and ORP8 localize to the plasma membrane with their PH domain and the preceding polybasic segment, corresponding to an α-helix (H1 in Fig. 2), that both interact with PI(4)P and phosphatidylinositol 4,5-bisphosphate [PI(4,5)P_2_] (Chung et al., 2015; Ghai et al., 2017; Guyard et al., 2022; Sohn et al., 2018). ORP8 is less efficient in localizing to the plasma membrane than ORP5. The negatively charged *N*-terminal IDR of ORP8 averts the plasma membrane, the inner leaflet of which is enriched in negatively charged lipids. Accordingly, ORP5 is more efficient in reducing plasma membrane PI(4)P levels than ORP8 (Sohn et al., 2018). The heterodimerization of ORP5 and ORP8 impedes the association of ORP5 with the plasma membrane due to the negatively charged IDR of ORP8. By this mechanism, dimerization maintains dynamic protein turnover at the ER–plasma membrane interface.

The ORP5–ORP8 dimer also connects the ER to mitochondria and lipid droplets (Du et al., 2020; Galmes et al., 2016; Guyard et al., 2022; Monteiro-Cardoso et al., 2022). ORP5–ORP8 localizes to these organelles by a different mechanism to that used for its interaction with the plasma membrane. ORP5 and ORP8 localization to mitochondria does not require their PH domain (Galmes et al., 2016). Unlike the plasma membrane localization, the dimerization of ORP5 and ORP8 promotes mitochondrial localization, further suggesting a regulatory role for LTP dimerization defining the subcellular localization of intracellular lipid trafficking (Galmes et al., 2016; Monteiro-Cardoso et al., 2022). At ER–mitochondria contact sites, the ORP5–ORP8 dimer mediates PS transport to the mitochondria for its conversion into phosphatidylethanolamine and regulates mitochondrial morphology and function.

Via an amphipathic α-helix found in the lipid transferring OSBP-related domain (ORD) of ORP5, the ORP5–ORP8 dimer also localizes to lipid droplets (Du et al., 2020; Guyard et al., 2022). The ORP5–ORP8 dimer recruits the ER protein seipin (BSCL2) to phosphatidic acid-enriched ER–mitochondria-lipid droplet tripartite contacts to regulate lipid droplet biogenesis in the ER (Guyard et al., 2022). As ORP8 shows a weaker association with lipid droplets, its association with this organelle increases by dimerization with ORP5.

### ORP9–ORP10 and ORP9–ORP11

**Fig. 4.**
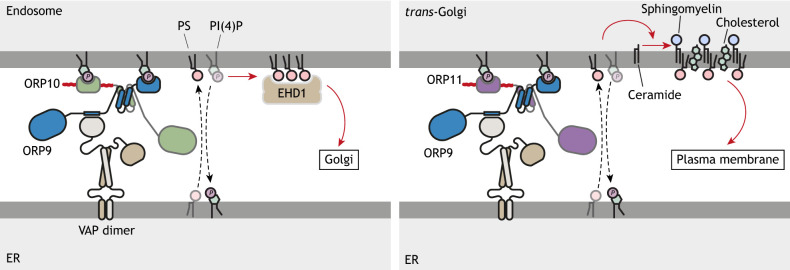
**ORP heterodimers at ER contact sites transform the lipid composition of opposing organelles.** The ORP9–ORP10 heterodimer localizes to the ER via the interaction between ORP9 and VAP proteins embedded into the ER membrane. Localization to the PI(4)P-rich t*rans*-Golgi is enhanced by two PH domains. PS transported by ORP9-ORP10 recruits the PS-binding EHD1 to endosomes, leading to endosomal fission for retrograde trafficking to the Golgi (left panel). The ORP9–ORP11 dimer localizes to the ER–*trans*-Golgi contact site in a analogous manner to the ORP9–ORP10 heterodimer (right panel). PS trafficking by ORP9–ORP11 promotes sphingomyelin synthesis at the *trans*-Golgi. The synchronization of PS transport and sphingomyelin synthesis at the ER–*trans*-Golgi contact site, together with lipid transfer by OSBP and CERT homodimers, define the lipid composition of the plasma membrane.

Despite their earlier prediction as PS transfer proteins, it is only recently that ORP9, ORP10 and ORP11 have been confirmed to transfer PS in exchange for PI(4)P between the ER and other membranes (Cabukusta et al., 2024; He et al., 2023; Kawasaki et al., 2022; Tan and Finkel, 2022). Unlike ORP5 and ORP8, ORP9 localizes to the ER via its FFAT (‘two phenylalanine residues in an acidic tract’) motif, which interacts with ER-resident VAP proteins. ORP10 and ORP11 achieve ER localization by dimerizing with ORP9. ORP9–ORP10 and ORP9–ORP11 dimerization is mediated by two α-helices in each protein (Figs 2 and 4) (Cabukusta et al., 2024; He et al., 2023; Naito et al., 2023; Zhou et al., 2010). Unlike OSBP and CERT, which contain one short and one long helix, ORP9, ORP10 and ORP11 have helices that are similar in length. Furthermore, ORP9 and ORP10 are unable to form homodimers (He et al., 2023; Tan and Finkel, 2022). Although each protein can exchange PS for PI(4)P, heterodimerization significantly enhances lipid transfer efficiency (Cabukusta et al., 2024; He et al., 2023).

Besides ORP10 and ORP11, ORP9 also benefits from dimerization, which facilitates its localization to organelles juxtaposed to the ER. As the ORP9–ORP10 and ORP9–ORP11 dimers connect the ER to the Golgi and endosomes, individual proteins fail to perform this function because their PH domains alone provide insufficient affinity for membrane association (Cabukusta et al., 2024; He et al., 2023; Kawasaki et al., 2022). The PH domains of ORP9 and ORP11 as a dimer display a stronger interaction with membranes, further underscoring the importance of dimerization (Ajiki et al., 2024; Cabukusta et al., 2024).

At ER–endosome contact sites, the ORP9–ORP10 dimer delivers PS to PI4KIIα-positive endosomes for endosome-to-Golgi retrograde trafficking (Fig. 4) (Kawasaki et al., 2022). PS on the endosomal membrane recruits the PS-binding dynamin-like ATPase EHD1, which mobilizes the retromer complex to promote endosomal fission for retrograde trafficking (Gokool et al., 2007). Consequently, loss of ORP10 delays the fission of SNX1-positive tubules from endosomes (Kawasaki et al., 2022). ORP11 is also localized at endosomes, but its function at these organelles remains less well studied (Arnal-Levron et al., 2019; Zhou et al., 2010).

At the ER–Golgi interface, PS–PI(4)P exchange mediated by the ORP9–ORP11 dimer promotes sphingomyelin synthesis in the Golgi (Fig. 4) (Cabukusta et al., 2024). Although loss of the ORP9–ORP11 dimer does not affect CERT activity – as shown by ceramide accumulation in the Golgi – it reduces the capacity of this organelle to synthesize sphingomyelin, suggesting that the correct phospholipid composition of the Golgi is crucial for sphingomyelin synthesis. Interestingly, ORP10 can function independently of ORP9 at the ER–Golgi interface (He et al., 2023; Venditti et al., 2019). ORP10-mediated PS levels in the Golgi sustain membrane contact sites between the ER and Golgi, whereas ORP9 or ORP11 do not play a role in this (Venditti et al., 2019).

In addition to their functions at various cellular interfaces in resting cells, ORP9, ORP10 and ORP11 support membrane homeostasis in response to lysosomal damage (Tan and Finkel, 2022). PI(4)P generated on the lysosomal delimiting membrane by permeabilizing damage recruits ORPs to this site (Radulovic et al., 2022; Tan and Finkel, 2022). Whereas ORP9–ORP10 and ORP9–ORP11 dimers, in complex with VAPA, are sufficient for membrane repair, individual ORPs cannot perform this function, highlighting the importance of LTP dimerization.

### OSBP–ORP4

ORP4 is the closest homolog to OSBP, and they share a similar domain architecture (Fig. 2) (Lehto et al., 2001). Regardless of their similarities, OSBP and ORP4 have different physiological roles. OSBP is ubiquitously expressed in all tissues and is essential for development, as OSBP knockout in mice is suggested to be embryonically lethal (Olkkonen and Ikonen, 2024). In contrast, ORP4 is mainly expressed in the brain, retina, heart, testes and macrophages, and its loss leads to male infertility (Jaworski et al., 2001; Udagawa et al., 2014; Wang et al., 2002; Zhong et al., 2016a).

**Fig. 5.**
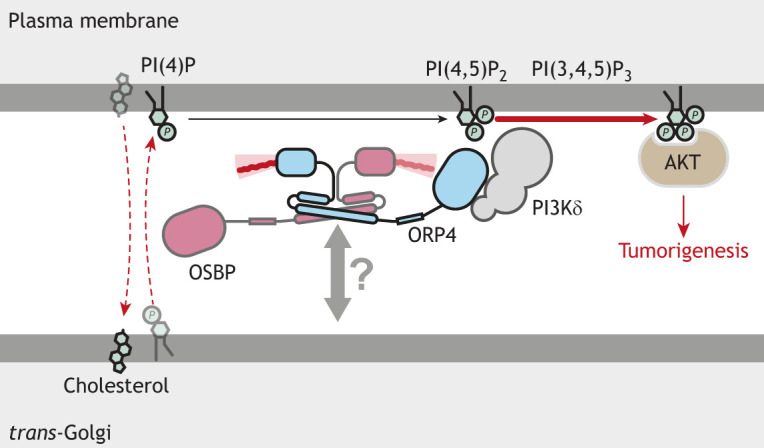
**PI(4)P transfer by the OSBP–ORP4 heterodimer from the *trans*-Golgi to the plasma membrane promotes the activation of the kinase AKT.** Ectopic expression of ORP4 in T cells recruits OSBP to the plasma membrane, where it transfers PI(4)P from the Golgi pools to the plasma membrane. The mechanism by which OSBP shuttles between these organelles remains unclear. Golgi-derived PI(4)P prevents exhaustion of PI(4,5)P_2_ on the plasma membrane. ORP4 stimulates PI3Kδ to synthesize PI(3,4,5)P_3_, which activates an AKT signaling pathway, promoting many processes, including cell growth and tumorigenesis.

The dimerization interface of OSBP is highly conserved in ORP4 (Wyles et al., 2007). Despite this, ORP4 is unable to homodimerize, but it can heterodimerize with OSBP (Fig. 5) (Wyles et al., 2007). This dimerization, likely mediated by coiled coils, drives ORP4 localization to ER–Golgi membrane contact sites where it regulates PI(4)P levels and Golgi structure (Pietrangelo and Ridgway, 2018).

Ectopic expression of ORP4 is found in leukemia T cells and leukemia stem cells, suggesting that it has an additional, carcinogenic role (Zhong et al., 2019, 2016b). In a xenograft mouse model, ORP4 is necessary for the survival of leukemia T cells. ORP4 localizes to the plasma membrane in T cells and recruits OSBP to the same site upon anti-CD3 stimulation, a treatment mimicking T cell activation (Fig. 5) (Zhong et al., 2022). As a result, OSBP transfers PI(4)P from the Golgi to the plasma membrane in exchange for cholesterol. Interestingly, lipid trafficking at the plasma membrane by the OSBP–ORP4 dimer occurs without the involvement of ER-localized VAP proteins. The mechanism by which the dimer shuttles between the Golgi and plasma membrane remains to be discovered.

PI(4)P transported by OSBP promotes phosphatidylinositol (3,4,5)-trisphosphate [PI(3,4,5)P_3_] generation at the plasma membrane through ORP4-activated phosphoinositide 3-kinase subunit δ (PI3Kδ, encoded by *PIK3CD*) (Zhong et al., 2022). PI(3,4,5)P_3_ activates AKT family proteins, which are kinases often hyperactivated in cancers (Revathidevi and Munirajan, 2019). The OSBP–ORP4 heterodimer activates AKT, resulting in T cell deterioration and leukemia formation (Zhong et al., 2022). Similarly, aberrant upregulation of ORP4 expression has been reported in tissues derived from individuals with ovarian cancer, and inhibiting ORP4 has been identified as a potential target for anti-cancer therapy (Bensen et al., 2021).

## Dimeric LTP adaptors mediate complex formation at contact sites

In addition to LTPs that dimerize at contact sites, their accessory proteins also dimerize. VAP proteins, which mediate ER localization by interacting with FFAT motifs in many LTPs, form homo- and hetero-complexes (Figs 3 and 4) (Cabukusta et al., 2020; Kim et al., 2010). This raises the question of the stoichiometry of LTP–VAP complexes. For LTP homodimers, one possibility is that each LTP interacts with a VAP from a different dimer, ‘zippering’ two organelles along the contact site. In this unfavorable scenario, the zippering effect is expected to impair LTP mobility and dynamics at contact sites, which are important for LTP function (as discussed for OSBP above). A more likely stoichiometry involves LTP–VAP complexes formed by dimer-to-dimer at contact sites. Some LTPs also contain double FFAT motifs, suggesting that a VAP dimer forms a complex with two motifs within an LTP (Mikitova and Levine, 2012). For example, two motifs in ORP3 contribute to its interaction with VAPA (Weber-Boyvat et al., 2015).

VAPA and VAPB also contain IDRs between the transmembrane and FFAT motif-targeting major sperm protein (MSP) domain, accommodating the dynamic nature of contact sites. These IDRs provide flexibility, functioning as molecular springs at sites where the intermembrane distance fluctuates (Subra et al., 2023b). For instance, transient contact sites like the ER–*trans-*Golgi contact require VAPA flexibility, whereas the flexibility provided by IDRs is not needed at more stable ER–mitochondria contact sites.

Transmembrane Emp24 domain-containing protein 2 (TMED2) and TMED10 are two other dimeric transmembrane adaptors present at ER–*trans*-Golgi contact sites (Fig. 3). The TMED2–TMED10 dimer tethers OSBP and CERT to the *trans-*Golgi and is essential for their interaction with VAPA (Anwar et al., 2022). TMED2–TMED10 absence reduces cholesterol-rich nanodomains on the plasma membrane (Anwar et al., 2022). By joining VAPA, VAPB, OSBP and CERT in a super-complex, the TMED2–TMED10 dimer supports the co-regulation of cholesterol and sphingolipid levels on the plasma membrane. As a part of this super-complex, OSBP and CERT likely localize to the same PI(4)P-rich subdomains within the *trans*-Golgi.

At the *trans-*Golgi, CERT interacts with PI(4)P pools of which production is dependent on the ARL5 effector protein DGARM (also known as C10orf76 and ARMH3) (Ishida et al., 2024; Mizuike et al., 2023). A genetic screen linking OSBP function to DGARM further supports the co-operation of ceramide and cholesterol trafficking at the ER–Golgi interface, suggesting that OSBP, similar to CERT, binds to DGARM-specific PI(4)P pools (Cigler et al., 2025). Moreover, CERT has been identified in the proximity landscape of OSBP (Kovacs et al., 2024). As PS transferred by ORP9–ORP11 to the Golgi promotes sphingomyelin synthesis, synchronization of anterograde PS trafficking with sphingomyelin synthesis is likewise important for maintaining the asymmetric lipid composition of the plasma membrane. The dimerization of LTPs and their adaptors plays a fundamental role in this.

## Conclusions and future directions

Non-vesicular trafficking of lipids by LTPs defines the lipid composition of cellular membranes. By controlling intracellular lipid distributions, LTPs also define the developmental fate and identity of cells, such as OSBP in epithelial cells and ORP4 in cancerous cells (Kovacs et al., 2024; Zhong et al., 2016b). LTP homo- and hetero-dimerizations mediated by coiled coils are pivotal to LTP function, as dimerization not only provides localization at membrane contact sites but also structural stability, making lipid transfer more adept.

Given that lipid trafficking events are highly dynamic, changes in the oligomeric or conformational state of LTPs would allow rapid regulation of intracellular lipid flow (Capasso et al., 2017; Radulovic et al., 2022; Tan and Finkel, 2022). For CERT, the kinase and phosphatase controlling its activity, and possibly dictating its dimeric conformation are defined (Saito et al., 2008; Tomishige et al., 2009). The regulation of dimerization of other LTPs – such as OSBP, ORP9, ORP10 and ORP11 – remains to be elucidated. OSBP is also phosphorylated at multiple sites, but the effects of these modifications on dimerization are yet to be defined (Goto et al., 2012). As OSBP-mediated cholesterol trafficking is downregulated during epithelial-to-mesenchymal transition, events controlling this transition could be in charge of OSBP dimerization and activity (Kovacs et al., 2024). Considering the genetic and physical interactions between sphingomyelin and cholesterol, the plasma membrane levels of these lipids are likely co-regulated. An interesting notion is that there is an interaction between OSBP and CERT via their shared coiled coil regions, which would allow synchronization of two lipid flows. Also, for the ORD family members ORP5, ORP8, ORP9, ORP10 and ORP11, their localization at various membrane contact sites is mediated by dimerization, and a switch between a monomer and a dimer has potential to dictate this localization; however, the lifetime and dynamics of these dimers have not been experimentally determined.

Many LTPs are shown to form dimers, but whether dimerization represents the last state of these self-assemblies remains an open question. Trimeric and tetrameric forms of CERT have been observed, and a secreted form of CERT has even been reported to form higher-order oligomers (Charruyer et al., 2008; Gehin et al., 2023; Goto et al., 2022; Raya et al., 2000; Revert et al., 2018). Despite these findings, most structural evidence for LTP dimers remains hypothetical. For instance, the dimerization of OSBP has been demonstrated using protein fragments (Fig. 2), leaving the physiological relevance of the full-length dimer unresolved (de la Mora et al., 2021). Similarly, it remains to be determined whether ORP9–ORP10 interacts with ORP9–ORP11 to form larger assemblies. Such a structure of four LTPs contains two FFAT motifs, matching the binding capacity offered by a VAP dimer. In the case of OSBP–ORP4 interaction, it is unclear whether ORP4 binds to a monomeric or a dimeric OSBP, further highlighting the need for structural insight.

This Review discusses only a subset of LTPs, but dimerization is not limited to these proteins, nor is the dimerization mechanism restricted to coiled coils. For instance, ORP1 (also known as OSBPL1A) forms dimers and trimers using its lipid transfer domain, and ORP2 (also known as OSBPL2) forms tetramers similarly (Dong et al., 2019; Wang et al., 2019). Furthermore, ORP1, which is involved in PI(4)P-mediated membrane repair after lysosomal damage, can dimerize via the lysosomal GTPase Rab7 proteins, which is turn are dimerized through its effector protein RILP (Johansson et al., 2007; Ma et al., 2018; Radulovic et al., 2022; Rocha et al., 2009). Many other ORPs and LTPs contain coiled coils. Only recently have we begun to understand the biophysical, biological and clinical significance of LTP dimerization. It is likely that the oligomeric state and stoichiometry of other LTPs and membrane contact site proteins play a similarly important role in lipid flows that define organelle lipid landscapes and cellular fate.
